# Supplementary material for: Genome-Wide Identification of Auxin Response Factors in Peanut (Arachis hypogaea L.) and Functional Analysis in Root Morphology
Source: Int J Mol Sci. 2022 May 10;23(10):5309. doi: 10.3390/ijms23105309 (PMC9141974; doi:10.3390/ijms23105309)
Supplement: Supplementary file 1 [file ijms-23-05309-s001.zip › Figure S3 Multiple sequence alignment of AhARF1 (A) and AhARF63 (B) with other peanut ARFs.pdf]

Figure S3

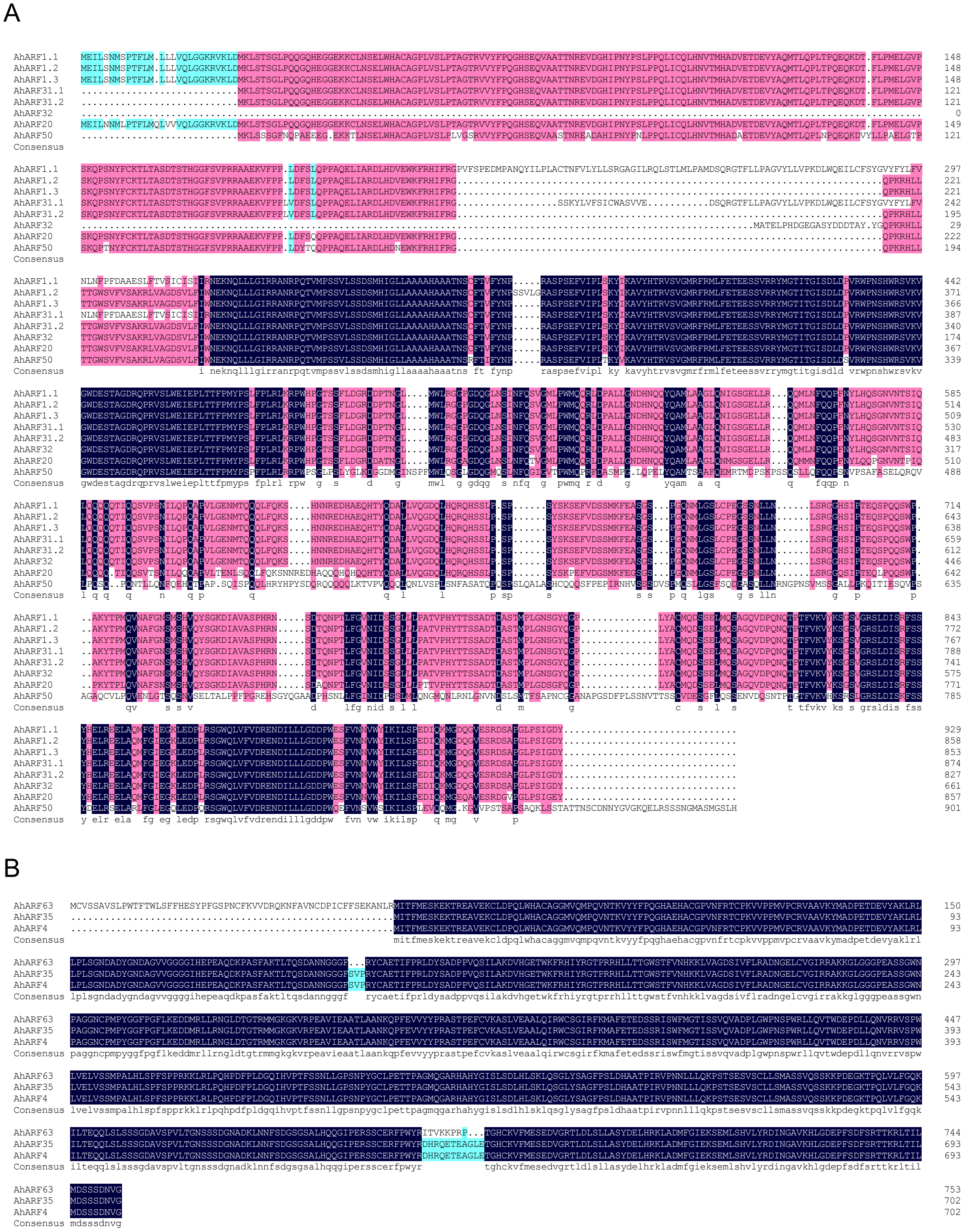

Figure S3 Multiple sequence alignment of AhARF1 (A) and AhARF63 (B) with other peanut ARFs. The correspondence between ARFs identified in this study and previous study are, *AhARF31* and *AhARF8-2*, *AhARF20* and *AhARF8-1*, *AhARF30* and *AhARF18-6*, *AhARF50* and *AhARF6-5*, *AhARF35* and *AhARF18-7*, *AhARF4* and *AhARF8-1*.
