# Supplementary material for: Genome-Wide Identification of Auxin Response Factors in Peanut (Arachis hypogaea L.) and Functional Analysis in Root Morphology
Source: Int J Mol Sci. 2022 May 10;23(10):5309. doi: 10.3390/ijms23105309 (PMC9141974; doi:10.3390/ijms23105309)
Supplement: Supplementary file 1 [file ijms-23-05309-s001.zip › Figure S4 Subcellular prediction of AhARF14 and AhARF26 using DeepLoc-1.0.pdf]

Figure S4

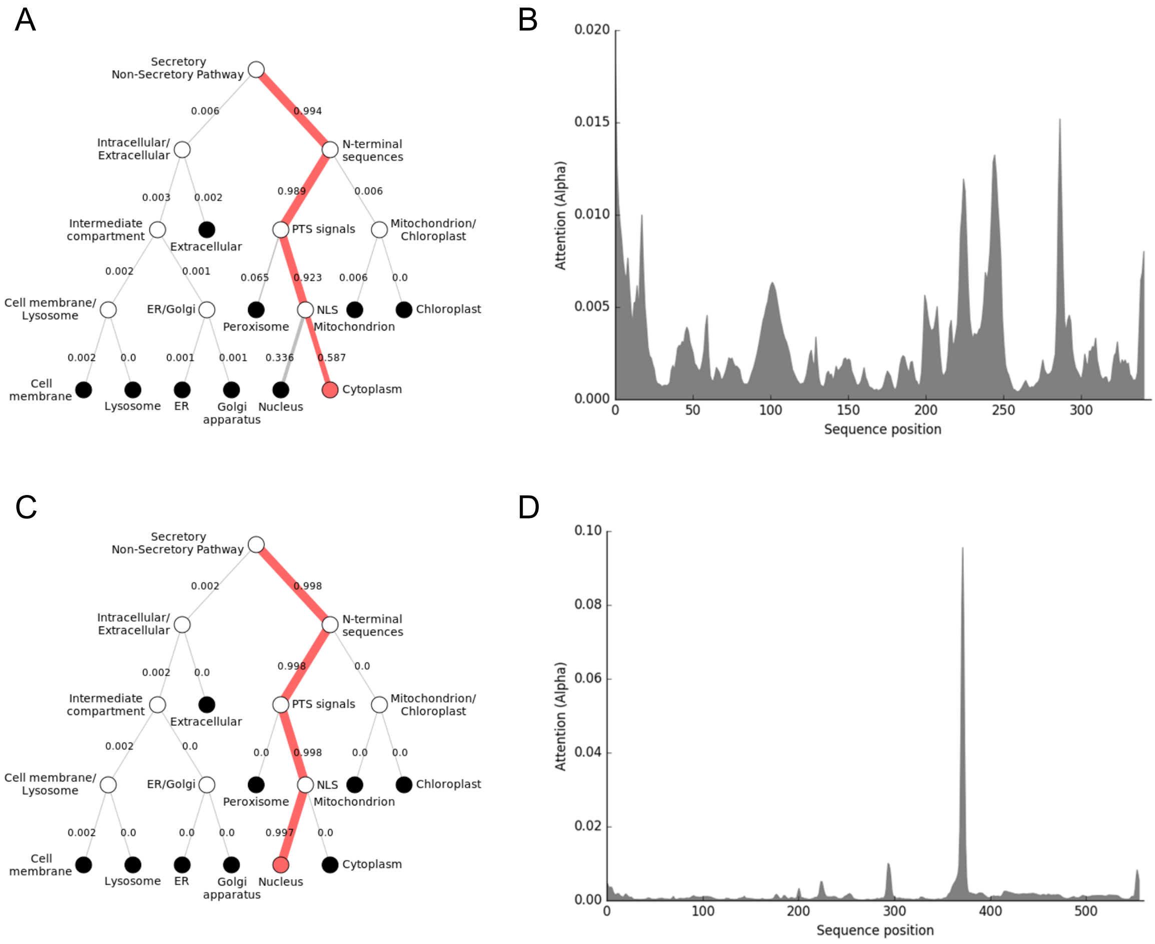

Figure S4 Subcellular prediction of AhARF14 and AhARF26 using DeepLoc-1.0. **(A)** Hierarchical tree and **(B)** alpha value of AhARF14; **(C)** Hierarchical tree and **(D)** alpha value of AhARF26.
