# Supplementary material for: Genome-Wide Identification of Auxin Response Factors in Peanut (Arachis hypogaea L.) and Functional Analysis in Root Morphology
Source: Int J Mol Sci. 2022 May 10;23(10):5309. doi: 10.3390/ijms23105309 (PMC9141974; doi:10.3390/ijms23105309)
Supplement: Supplementary file 1 [file ijms-23-05309-s001.zip › Table S5 Botanical type and origin of 11 peanut varieties.pdf]

Table S5 Botanical type and origin of 11 peanut variaties

Germplasm Name	Botanical Type	Origin	Remarks
Jinkins Jumbo	var. *hypogaea*	America	
Meiyinxuan41159	var. *hypogaea*	America	
PI295250	var. *hypogaea*	Israel	
PI504614	var. *hypogaea*	Colombia	
PI648250	var. *aequatoriana*	Ecuador	
Shixuan64	*var. vulgaris*	China	
Juhua27	*irregular*	China	
Fenghua2	var. *vulgaris*	China	Main cultivar
Ehua2	*irregular*	China	Main cultivar
Shanhua11	*irregular*	China	Main cultivar
Zhonghua12	var. *vulgaris*	China	Main cultivar
