# Supplementary figures and images for: Genome-Wide Identification of Auxin Response Factors in Peanut (Arachis hypogaea L.) and Functional Analysis in Root Morphology
Source: Int J Mol Sci. 2022 May 10;23(10):5309. doi: 10.3390/ijms23105309 (PMC9141974; doi:10.3390/ijms23105309)

Figure S1

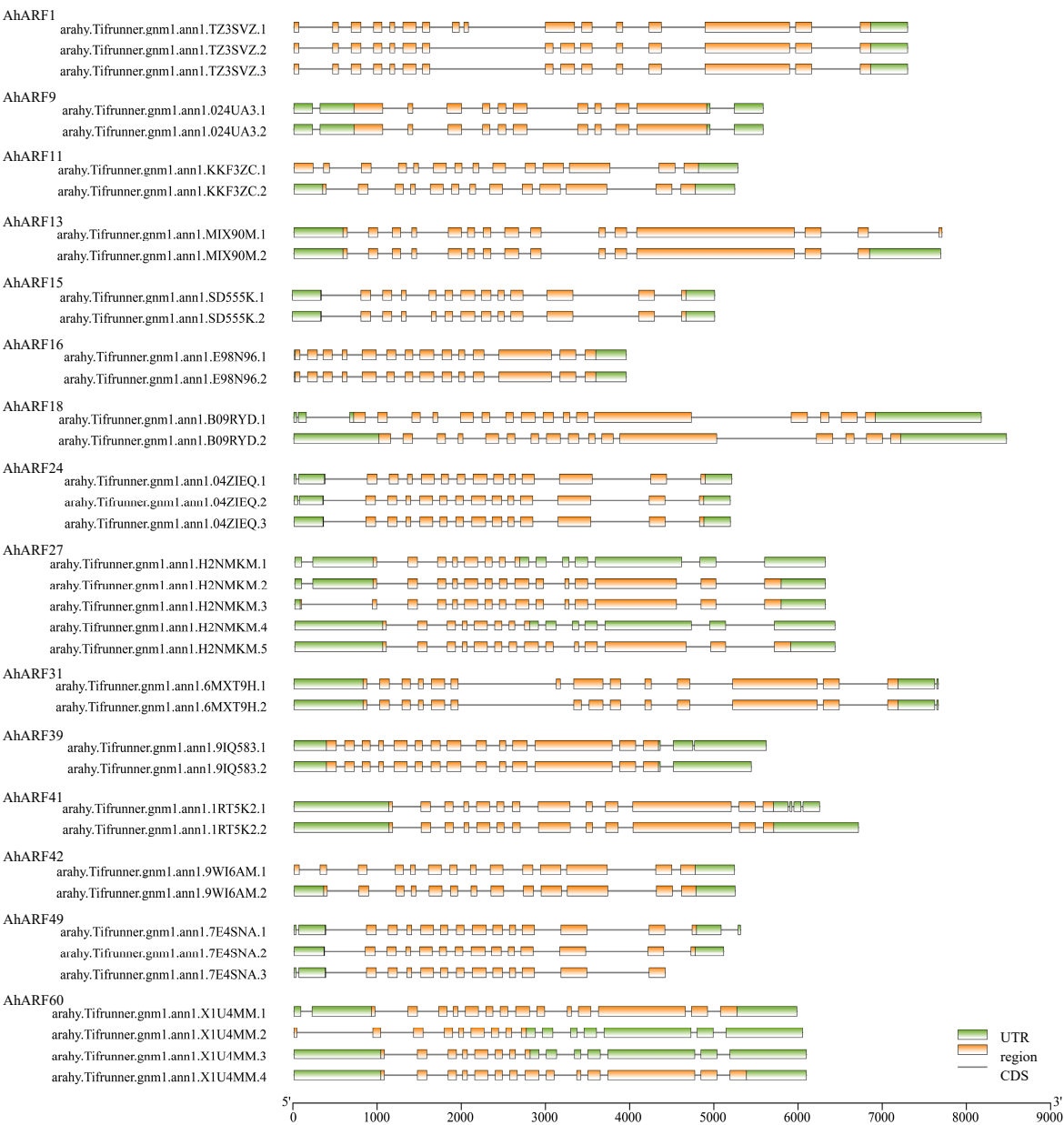

Figure S1 Structure analysis of *AhARFs* with alternative splicing events.

Supplement: Supplementary file 1 [file ijms-23-05309-s001.zip › Figure S1 Structure analysis of AhARFs with alternative splicing events.pdf]

**Figure S2**

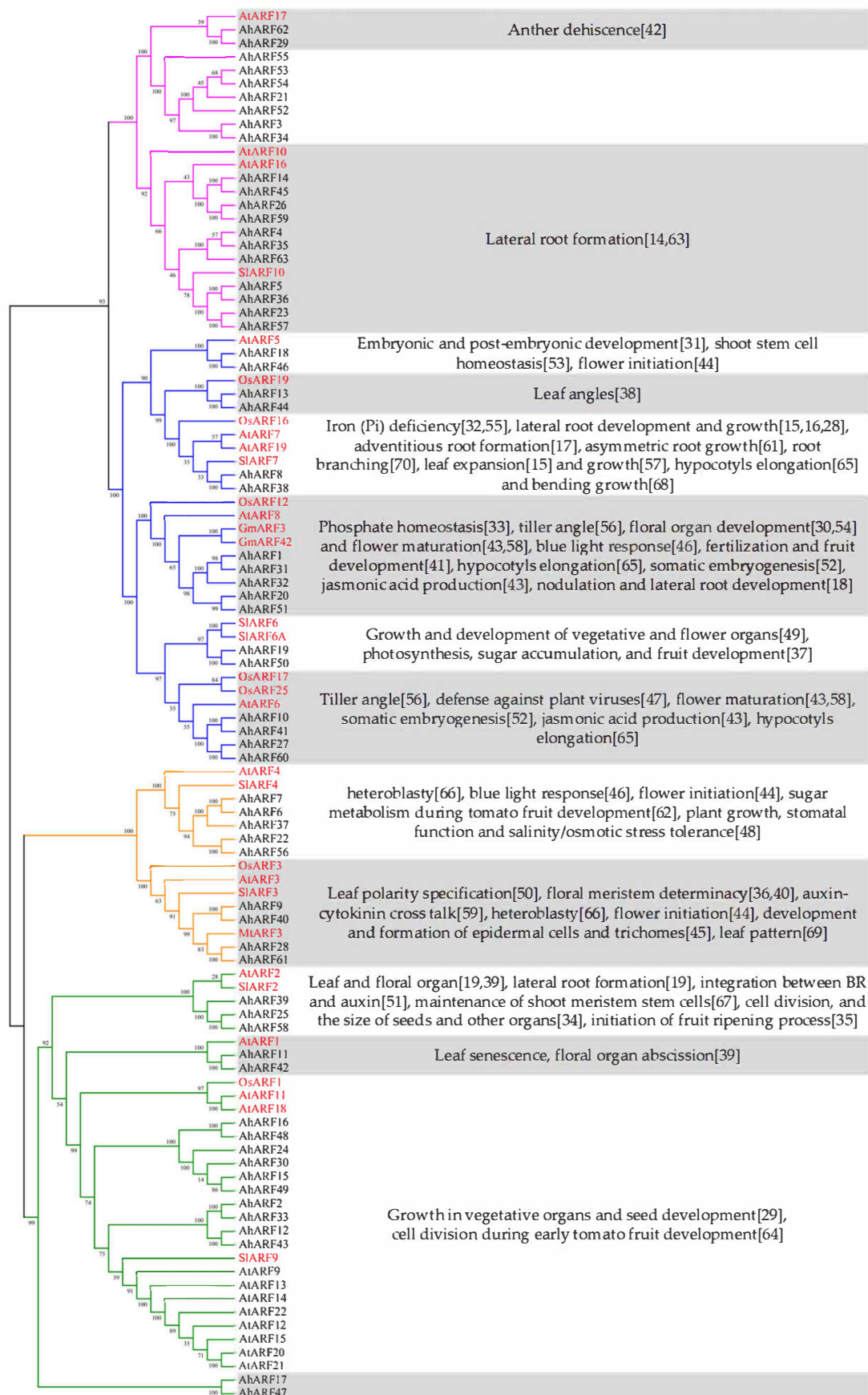

**Figure S2. Phylogenetic analysis and function prediction of plant ARFs**

Supplement: Supplementary file 1 [file ijms-23-05309-s001.zip › Figure S2 Phylogenetic analysis and function prediction of plant ARFs.pdf]
